# Comparison of Social Inequality in Human Papillomavirus (HPV) Vaccination among Teenagers with Parental Reports and Healthcare Providers’ Records in the 2019 National Immunization Survey-Teen

**DOI:** 10.3390/vaccines10020178

**Published:** 2022-01-24

**Authors:** Sol Seo Choi, BongKyoo Choi

**Affiliations:** 1 University High School, Irvine, CA 92612, USA; sol.seo.choi@gmail.com; 2 Center for Work and Health Research, 35 Schubert Court, Irvine, CA 92617, USA; 3 Department of Medicine, University of California, Irvine, CA 92617, USA

**Keywords:** human papillomavirus, vaccine, social inequality, NIS-Teen, false negative, SES

## Abstract

Background: Relatively little is known about social inequality in human papillomavirus (HPV) vaccination among teenagers in the United States. This study aims to investigate whether there is a social disparity in HPV vaccination among teenagers and if so, whether it can differ according to the source of teen vaccination information (parental reports and provider records). Methods: We used the data from the 2019 National Immunization Survey-Teen (NIS-Teen; 42,668 teenagers, aged 13–17) including parent-reported vaccination status. Among them, 18,877 teenagers had adequate provider-reported vaccination records. Two socioeconomic status (SES) measures were used: mother’s education and annual family income. Multivariate logistic analyses were conducted. Results: False negatives of parental reports against provider records were more than two times higher (*p* < 0.001) in low-SES teens than in high-SES teens. In both SES measures, the proportion of HPV-unvaccinated teenagers was lowest at the highest SES level in analyses with parental reports. However, it was the opposite in analyses with provider records. Interestingly, regardless of the vaccination information source, the HPV unvaccinated rate was highest in the middle-SES teens (>12 years, non-college graduates; above poverty level, but not >USD 75 K). Conclusions: Significant social inequality in HPV vaccination among teenagers exists in the United States. The pattern of social inequality in HPV vaccination can be distorted when only parent-reported vaccination information is used.

## 1. Introduction

Human papillomavirus (HPV) infection, resulting in cervical and other cancers, is the most common sexually transmitted disease (STD) [1]. While most HPV cases result in genital warts that may go away in time, persistent HPV infections have been the leading cause of “90.6% of cervical, 91.1% of anal, 75.0% of vaginal, 70.1% of oropharyngeal, 68.8% of vulvar, 63.3% of penile, 32.0% of oral cavity, and 20.9% of laryngeal cancers” [2] (p. 1). HPV infections have caused nearly 46,143 cancers each year from 2014 to 2018, affecting nearly 25,719 women and 20,424 men per year [3].

In the United States, HPV inactivated vaccines, such as Gardasil (quardrivalent), Cervarix (bivalent), and Gardasil 9 (nonavalent), have been developed for the prevention of HPV-associated cervical and other cancers, but since 2016, only Gardasil 9 has been distributed, targeting seven high-risk and two low-risk HPV types [4]. As of now, from ages 9 through 15, HPV vaccines are recommended to be administered in a two-dose series, while from ages 15 to 45 years old, a three-dose series is recommended. The evidence for the effectiveness (against cervical precancers) and safety of the HPV vaccine has been strong in high-quality well-designed studies [5,6,7]. A recent study also reported that “…From the prevaccine era to 2015–2018, significant decreases in 4vHPV-type prevalence occurred among females aged 14–19 years (88%) and 20–24 years (81%)” [8]. In addition, Falcaro et al. [9] demonstrated the effectiveness of the national HPV vaccination program for the prevention of cervical cancer as well as cervical precancers in young British women.

However, the HPV vaccination of teenagers and its promising future benefits may vary significantly between SES strata. Monitoring social inequality in HPV vaccination among teenagers while working towards the health equity of HPV vaccination is a primary national task for public health in the United States (US) [10].

Several investigators [11,12,13,14,15] have examined social inequality in HPV vaccination among teens, particularly using the National Immunization Survey (NIS) Teen, designed to generate accurate, nationally representative information on the vaccination coverage (including HPV vaccine) of teens in the US. However, the results of the previous studies have been inconclusive. For example, in [11,12], using parent-reported vaccination status, the HPV vaccination rate was greater in high-socioeconomic status (SES) teens than in low-SES teens. In contrast, in one study [13], using the provider-reported vaccination status, the HPV vaccination rate was greater in low-SES teens than in high-SES teens. It is not yet clear why they came to different conclusions about social inequality in HPV vaccination among teens, although both studies were based on the same data source of the NIS-Teen. 

However, several studies [16,17,18,19] have analyzed discrepancies between parent-reported vaccination status and provider-reported vaccination records, which may be biased towards either severe overestimation or underestimation, depending on the type of disease-preventing vaccine. Dorell et al. [16] reported that more than 20% of parent-reported HPV vaccination status of teens in the 2008 NIS-Teen data were false-negative against provider-reported vaccination records. Ojha et al. [17] further demonstrated that the false-negative propensity of parent-reported vaccination status was greater in low-SES teens than in high-SES teens. All of this implies that social inequality in HPV vaccination among teens may significantly differ according to which source of vaccination information in the NIS-Teen is used [18]. However, no studies have examined and compared social inequality in HPV vaccination by using both the parent- and provider-reported vaccination information of the NIS-Teen. 

This study aimed to investigate (1) whether there is a significant difference in HPV vaccination according to SES among teens and if so, (2) whether it differs according to the source of teen vaccination information (parental reports and provider records) in the recent 2019 NIS-Teen data of the US. 

## 2. Materials and Methods 

### 2.1. The National Immunization Survey-Teen (NIS-Teen) Data

The NIS-Teen was launched in 2006 and is sponsored by the National Center for Immunization and Respiratory Diseases of the Centers for Disease Control and Prevention (CDC). It has been used to monitor vaccination coverage among teens aged 13–17 years living in the US [20]. The NIS-Teen, an annual nationwide survey, uses two phases of data collection to obtain vaccination information for a large nationally representative sample of teens aged 13–17 years: (1) a random digit dialing (RDD) telephone survey of households with teens 13–17 years (“parental-reported vaccination information of teens”), followed by (2) a mailed immunization history survey to teens’ vaccination providers (“provider-reported vaccination information of teens”). The RDD survey was designed to identify households with 13–17-year-old teens. Once the households were identified, one parent (parent or guardian) per household who was most knowledgeable about the vaccination history of his/her eligible teen was interviewed by telephone. At the end of the interview, the parent was asked to provide oral consent for contacting his/her eligible teen’s vaccination providers. With parental oral consent, a questionnaire was mailed to each vaccination provider to collect the information regarding the types of vaccinations, number of doses, dates of administration, and other administrative data about the health care facility [20]. 

### 2.2. The 2019 NIS-Teen Data (N = 42,668)

**Figure 1:**
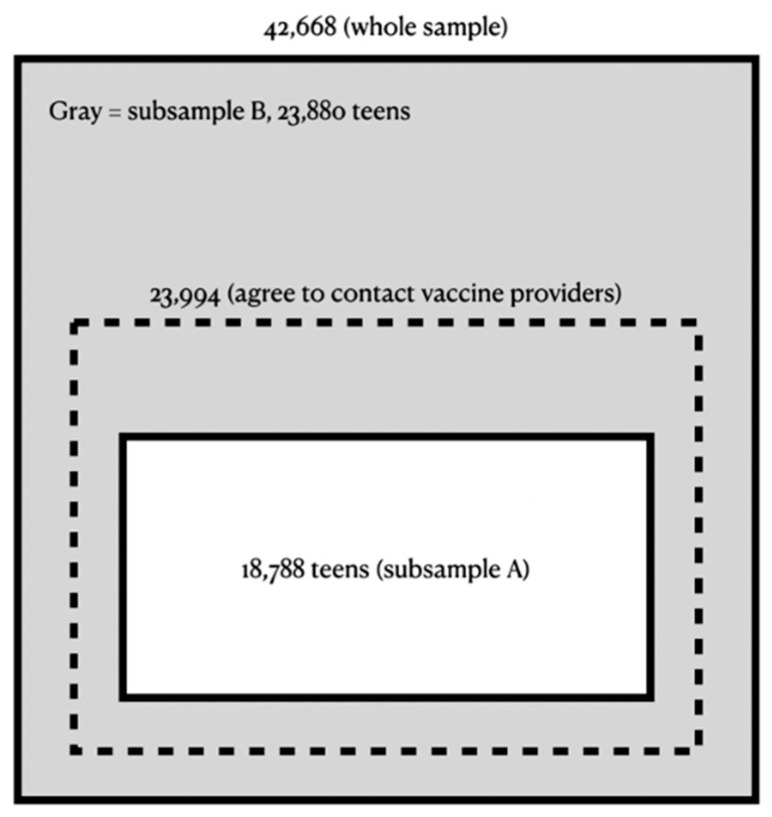
Study samples for the current study: the whole sample (42,668 teens) and the two subsamples, subsample A (18,788 teens) and subsample B (23,880 teens).

For the current study, we used the public-use data file from the 2019 NIS-Teen since it is the latest one available to the public for research prior to the full-scale coronavirus disease 2019 pandemic. For the 2019 NIS-Teen, the RDD household phone interview was conducted from 3 January 2019 to 6 February 2020, while data from teens’ vaccination providers were collected from January 2019 to March 2020. A total sample of approximately 17.6 million telephone numbers (excluding the territory samples) yielded 59,721 eligible parents with teens aged 13–17 years for the RDD phone interview. Among them, 42,668 parents completed the phone interview about their teen’s vaccination history (the whole sample). Additionally, among those who completed the RDD phone interview, 23,994 parents (56.2%) agreed to contact the vaccination providers. Finally, among those who completed the RDD phone interview, 18,788 teens (44.0%, “Subsample A”) were determined to have adequate provider data, which means “sufficient vaccination history information was obtained from the provider(s) to determine whether the teen is up-to-date with respect to the recommended vaccination schedule” [12] (p. 8). As a result, 23,880 teens of the whole sample (56.0%, “Subsample B”) were not included in Subsample A (Figure 1). For the analyses of the current study, the two subsamples, as well as the whole sample of 42,668 teens, were used. 

### 2.3. HPV-Unvaccinated Teens

The HPV vaccination status of teens was assessed with the parent-reported vaccination information (Method 1) in the whole and Subsample A. Parents responded to one interview question (“Has [teen name] ever received any human papillomavirus shots?”) with four response options (Yes; No; Don’t Know; and Refused). HPV-unvaccinated teens were defined as teens whose parents responded to the interview question with “No”. In addition, the HPV vaccination status of teens was assessed with the provider-reported vaccination information (Method 2) in Subsample A. The number of HPV shots, which was determined from the vaccination provider data, was available in the NIS-Teen data. HPV-unvaccinated teens were defined as teens whose vaccination providers reported no HPV vaccination. 

### 2.4. Socioeconomic Status of Teens

Teens’ SES was measured with two indicators: mother’s education and annual family income. Each indicator was measured with a single interview question. 

### 2.5. Covariates

The following teen-related covariates were considered in the analyses of the current study: age, sex, geographical region, and race/ethnicity. Each of the covariates was measured with a single interview question. 

### 2.6. Statistical Analyses

The distributions of sociodemographic characteristics and HPV vaccination status of teens were first examined in the whole sample. Then, the distributions were compared between the two subsamples using the Pearson chi-square test. In addition, we compared the parent-reported vaccination response against the provider-reported vaccination record (the reference) in Subsample A, which was also replicated by each level of SES due to potential SES-differential false negative percentages [17,18,19]. The distributions of the proportion of HPV-unvaccinated teens were examined by two SES indicators in the whole sample (Method 1) and Subsample A (both Methods 1 and 2). Utilizing multivariate logistic models, the distributions of the proportion of HPV-unvaccinated teens by two SES indicators were further examined after controlling for age, sex, race/ethnicity, and region. Considering the complex sampling design of the 2019 NIS-Teen, the aforementioned analyses were replicated using the SPSS 28.0 version of complex samples function. The results were very similar to those with unweighted data. For simplicity, we present here only the results with the unweighted 2019 NIS-Teen data.

## 3. Results

### 3.1. Sociodemographic Distributions of the Teens in the Whole Sample and the Two Subsamples

Table 1 shows the distributions of age, sex, race/ethnicity, region, mother’s education, and annual family income in the whole sample and the two subsamples. There were significant differences in all sociodemographic variables, except sex, between the two subsamples. In general, about 50% of the teens’ mothers were college graduates, while about 10% of the teens’ mothers had less than 12 years of formal education years across the whole sample and three subsamples. Annual family income was also greater than USD 75,000 in about 50% of the teens, while it was below the poverty level in about 10% of the teens. 

### 3.2. Vaccination Status of the Teens in the Whole Sample and the Two Subsamples 

In the whole samples, 25,263 (59.2%) out of 42,668 parents responded to the any HPV shot question with “Yes”; 32.1% with “No”; 8.6% with “Don’t Know”; and 0.1% with “Refused” (Table 1). The distribution of the parental responses was generally similar in the two subsamples, although the response with “Yes” increased in Subsample A (64.3%), while it decreased in Subsample B (55.2%). 

### 3.3. Comparison of the Parent- and Provider-Reported Vaccination Information in Subsample A 

**Figure 2:**
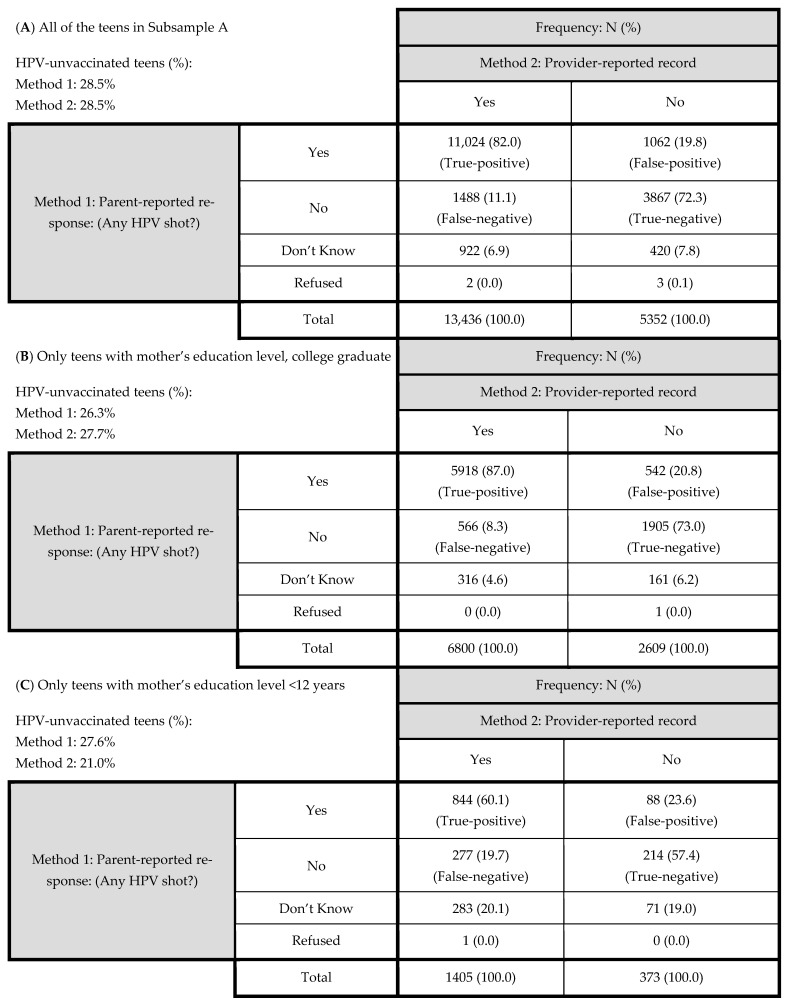
Comparison of the parent-reported responses (Method 1) against the provider-reported vaccination records (Method 2: the reference) for (**A**) all of the teens in Subsample A (N = 18,788), (**B**) only teens with mother’s education level (college graduate) (N = 9409), and (**C**) only teens with mother’s education level (<12 years) (N = 1778) of Subsample A. HPV: human papillomavirus. False negative cases are significantly higher in Figure 2C (19.7%) than in Figure 2B (8.3%). Additionally, a greater difference in the proportion of unvaccinated teens between Methods 1 and 2 can be observed in Figure 2C (6.6%) than in Figure 2B (1.4%).

The parent responses to the HPV shot question were compared with the provider information on teen’s HPV vaccination in Subsample A. Figure 2A shows that the true-positive percentage of the parental response against the provider information was 82%, while the true-negative percentage was 72.3%. The false-negative percentage (“No” response in the parental report, but ≥1 shot of HPV in the provider record) was 11.1%, while the false-positive percentage (“Yes” response in the parental report, but no shot of HPV in the provider record) was 19.8%. Out of the 18,788 teens in Subsample A, 2550 teens (13.6%) were false-negative or false-positive cases.

When the analysis was replicated at each level of two SES indicators in Subsample A, a significant SES differential pattern was observed. The proportions of the false-negative and false-positive cases were highest in the teens with the lowest SES level, while it was lowest in the teens with the highest SES level (*p* < 0.001): for example, 20.5% vs. 11.8% with mother’s education; 20.2% vs. 11.4% with annual family income (Table 2). In particular, the proportion of false-negative cases was more than two times higher in the teens with the lowest SES level than in the teens with the highest SES level (Table 2). For example, according to mother’s education level, the proportion of false-negative cases was higher in the teens with the lowest level than in the teens with the highest level of mother’s education: 19.7% vs. 8.3% (*p* < 0.001) (Figure 2B,C). As a result, the proportion of HPV-unvaccinated teens with Method 1 was slightly greater in the lowest SES teens than in the highest SES teens in terms of mother’s education level: 27.3% vs. 26.3%. However, with Method 2, it was reversed: 21.0% vs. 27.6% (Figure 2B,C).

### 3.4. HPV-Unvaccinated Teens (Method 1) According to SES in the Whole Sample 

The proportion of HPV-unvaccinated teens (Method 1: parent-reported) was lowest in the teens with the highest SES level with both mother’s education and annual family income in the whole sample (Table 3). However, the differences between SES strata were marginal: 4.5 percentage points for mother’s education and 2.6 percentage points for annual family income. Interestingly, the proportion of HPV-unvaccinated teens was highest in the teens with the middle-SES levels (>12 years, non-college graduates; above poverty level, but not greater than USD 75 K). On the other hand, parent “Yes” responses to the interview question significantly varied according to mother’s education level (63.7% vs. 47.5%), and “Don’t Know” responses were inversely associated with mother’s education levels (6.3% vs. 21.5%). Similar patterns were observed with annual family income in the whole sample (Table 3). 

### 3.5. HPV-Unvaccinated Teens (Method 1 and Method 2) According to SES in Subsamples A 

**Figure 3:**
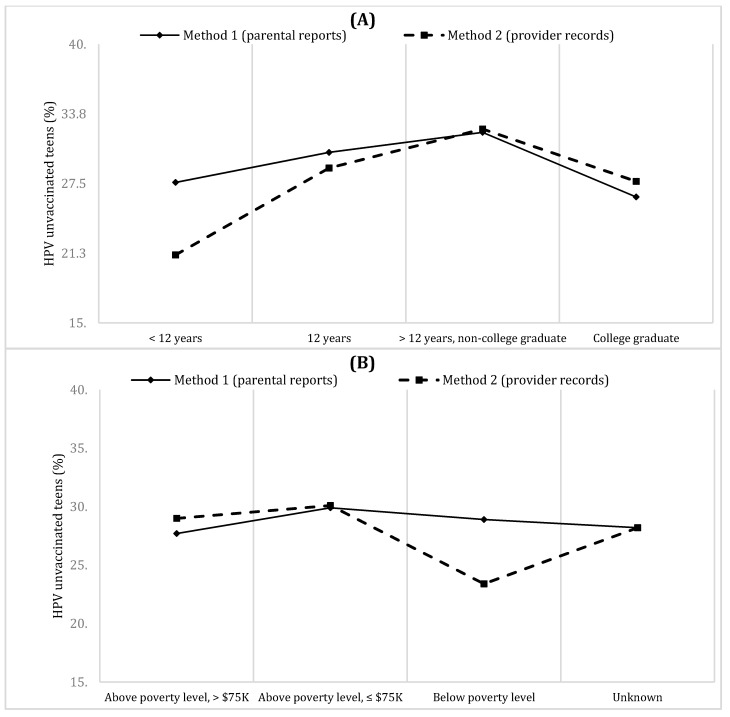
The distributions of the proportion of HPV-unvaccinated teens according to (**A**) mother’s education and (**B**) annual family income in Subsample A (N = 18,788). Method 1: parent-reported vaccination status. Method 2: provider-reported vaccination record. HPV: human papillomavirus. The biggest differences in the proportion of unvaccinated teens between Methods 1 and 2 were observed in the lowest SES groups (<12 years and below poverty level).

In Subsample A, the distributions of HPV-unvaccinated teens (Method 1) according to mother’s education and annual family income were similar to in the whole sample (Table 4). When Method 2 (parent-reported) was applied in Subsample A, a different pattern was observed (Table 5 and Figure 3): the proportion of HPV-unvaccinated teens was lowest in the teens with the lowest SES level, while it was highest in the teens with middle-SES levels (6.6 percentage points difference with mother’s education and 5.6 percentage points difference with annual family income).

### 3.6. Multivariate Analyses of HPV-Unvaccinated Teens According to SES in the Whole Sample and Subsample A

Table 6 shows the results of multivariate analyses for HPV-unvaccinated teens according to SES after controlling for age, sex, race/ethnicity, and region in the whole sample (Method 1) and Subsample A (Method 1 and Method 2). The results were consistent with those in the univariate analyses above. In the whole sample and Subsample A, the multivariate odds of HPV-unvaccinated teens (Method 1) was slightly, albeit statistically significant, greater in the teens with the lowest SES level than in the teens with the highest level of mother’s education or annual family income: odds ratios (ORs); 1.11~1.12 vs. 1.00; and 1.05~1.10 vs. 1.00, respectively. However, the pattern of the multivariate odds of HPV-unvaccinated teens according to SES (Method 2) in Subsample A was reversed. It was lower in the teens with the lowest SES level than in the teens with the highest level of mother’s education or annual family income: ORs, 0.81 and 0.84 vs. 1.00, respectively. 

Nonetheless, regardless of Method 1 and Method 2, the odds of HPV-unvaccinated teens were highest in the teens with the middle level of SES levels (>12 years, non-college graduates; above poverty level, but not greater than USD 75 K): ORs (compared to the teens with the highest SES level), 1.27~1.33 and 1.10~1.12, respectively. Additionally, as expected, in the multivariate logistic models, a significantly lower number of younger teens, who were 13–14 years old (vs. teens aged 17 years), male, non-Hispanic white, and non-Northeastern were vaccinated against HPV. 

## 4. Discussion 

To the best of our knowledge, this was the first study demonstrating that the direction of social inequality in HPV vaccination along the SES strata of teens can be reversed, depending on the source of teen vaccination information in the NIS-Teen data. In both SES measures, the proportion of HPV-unvaccinated teens was lowest in the highest SES level in analyses with parent-reported vaccination status. In contrast, it was lowest in the lowest SES level in analyses of provider-reported vaccination status. The false negative propensity of provider-reported vaccination status against provider vaccination records was greatest in the lowest SES level, which was the reason for the contrasting pattern of social inequality in HPV vaccination between the lowest and highest SES levels in the NIS-Teen data. Nonetheless, regardless of the source of teen vaccination information, the proportion of HPV-unvaccinated teens was highest in the teens with the middle-SES level (>12 years, non-college graduates; above poverty level, but not greater than USD 75 K). 

Our finding on social inequality in HPV vaccination with parent-reported vaccination information is consistent with two previous studies with data from the NIS-Teen [11,12]. However, the previous studies neither used provider-reported data nor considered SES-differential false-negative parent-reported vaccination information, which was observed in the current study as in the previous validation studies of the NIS-Teens [17,18,19]. Our finding on social inequality in HPV vaccination with provider-reported vaccination records is also consistent with the previous study [13]. Walker et al. [13] showed that the HPV vaccination rate was higher at the below poverty level than at or above the poverty level of teens using data from the 2018 NIS-Teen. However, we think that our finding is more robust because it was confirmed with not only annual family income, but also mother’s education and after controlling for several potential confounders. In addition, in the study by Walker et al. [13], a two-group comparison was performed (below the poverty level or not) rather than a three-group comparison of annual family incomes, as in the current study, so it could not detect any significant difference in HPV vaccination between the two groups (>USD 75 K or not) at or above the poverty level. 

This study has several important implications for research and public health policies on social inequality in HPV vaccination among US teens. First, despite the increasing rates of HPV vaccination in recent years [13,21], our study highlighted that HPV vaccination rates continue to be disparate according to the SES status of teens. There is no evidence that social inequality in cervical cancer mortality has been reduced over the past six decades [22]. The HPV vaccination of teenagers, as a major public health tool for the prevention of cervical and other cancers among teenagers, should be equitably administered, regardless of the socioeconomic background of teenagers. Second, in our study, non-linear social inequality in HPV vaccination among US teens was observed. Interestingly, we observed that the odds of HPV-unvaccinated teens in the extreme ends of the SES spectrum (of both mother’s education and annual family income) were in fact lower than those in the middle-SES categories. A similar result was observed in the two previous studies with provider-reported vaccination records in the 2012–2013 and 2016–2017 NIS-Teen [14,15], while some non-US studies reported a clear social gradient in HPV vaccination [23,24]. More in-depth studies [25] are needed to confirm and elucidate the underlying reasons for the non-linear social inequality in HPV vaccination among US teens in the future. If further confirmed in future studies, this may imply that instead of focusing solely on the extreme ends of the SES spectrum, public health policy makers need to ramp up HPV vaccination among teens in middle-SES categories, who may be comparatively lagging behind teens of high-end and low-end SES. Third, this study underscored the importance of using provider-reported vaccination records over parent-reported vaccination information regarding social inequality research on HPV and other vaccines using the NIS-Teen data. Otherwise, studies with only parent-reported vaccination status (Method 1) in the NIS-Teen data may lead to erroneous conclusions due to possible SES-differential false-negative propensity. Fourth, on the other hand, a future widespread use of CDC immunization information systems in the US may function as a tool to increase the quality of teen vaccination information from parents or healthcare providers, leading to more accurate and inclusive data, statistical analyses, and trends that can dictate future trends to decrease social inequality in teen HPV vaccination rates. 

Our study has some limitations. In our study, we utilized only a single year of data from the NIS-Teen due to our main focus on the methodological issues in the current study. Thus, the findings of our study remain to be confirmed in future studies with recent multi-year NIS-Teen data. In our study, about 44% of the whole sample (Subsample A) had adequate provider data. There were some significant differences in sociodemographic variables between Subsample A and Subsample B (56% of the whole sample). However, the results of our study were very similar in the whole sample and Subsample A with Method 1. The main difference in our findings was based on the method, not which subsample. We did not analyze the reasons for the observed nonlinear social inequality in HPV vaccination among US teens, which goes beyond the research question of the current study. Several researchers [14,25,26,27] have reported the most common parental reasons for not vaccinating teens against HPV in the US: safety concerns, lack of knowledge, not recommended by providers, not needed or not necessary, and not sexually active. Cost and time are not among the most common parental reasons for not vaccinating teens. We plan to examine any SES differential profiles of the most common parental reasons for non-intent to vaccinate their teens against HPV in the US in the near future [28]. 

## Figures and Tables

**Table 1:** Sociodemographic characteristics of the teens in the current study from the 2019 National Immunization Survey (NIS)-Teen.

Category	Sub-Category	Frequency: N (%)		
		In the Whole Sample (Total N = 42,668)	In Subsample A (Total N = 18,788)	In Subsample B (Total N = 23,880)
Age	13 years	8500 (19.9)	3927 (20.9) *	4573 (19.1) *
	14 years	8789 (20.6)	4007 (21.3) *	4782 (20.0) *
	15 years	8635 (20.2)	3753 (20.0) *	4882 (20.4) *
	16 years	8703 (20.4)	3753 (20.0) *	4950 (20.7) *
	17 years	8041 (18.8)	3348 (17.8) *	4693 (19.7) *
Sex	Men	22,293 (52.2)	9872 (52.5)	12,421 (52.0)
	Women	20,375 (47.8)	8916 (47.5)	11,459 (48.0)
Race/Ethnicity	Hispanic	8168 (19.1)	3466 (18.4) *	4702 (19.7) *
	Non-Hispanic White	26,014 (61.0)	11,883 (63.2) *	14,131 (59.2) *
	Non-Hispanic Black	3730 (8.7)	1365 (7.3) *	2365 (9.9) *
	Non-Hispanic Other and Mixed Race	4756 (11.1)	2074 (11.0) *	2682 (11.2) *
Region	Northeast	8107 (19.0)	3597 (19.1) *	4510 (18.9) *
	Midwest	8623 (20.2)	3985 (21.2) *	4638 (19.4) *
	South	16,945 (39.7)	7057 (37.6) *	9888 (41.4) *
	West	8993 (21.1)	4149 (22.1) *	4844 (20.3) *
Mother’s education	<12 years	4107 (9.6)	1778 (9.5) *	2329 (9.8) *
	12 years	6585 (15.4)	2680 (14.3) *	3905 (16.4) *
	>12 years, non-college graduate	11,199 (26.2)	4921 (26.2) *	6278 (26.3) *
	College graduate	20,777 (48.7)	9409 (50.1) *	11,368 (47.6) *
Annual family income	Above poverty level, >USD 75 K	21,891 (51.3)	10,061 (53.6) *	11,830 (49.5) *
	Above poverty level, ≤USD 75 K	11,954 (28.0)	5264 (28.0) *	6690 (28.0) *
	Below poverty level	5885 (13.8)	2803 (14.9) *	3082 (12.9) *
	Unknown	2938 (6.9)	660 (3.5) *	2278 (9.5) *
Ever any HPV shots?	Yes	25,263 (59.2)	12,086 (64.3) *	13,177 (55.2) *
	No	13,701 (32.1)	5355 (28.5) *	8346 (34.9) *
	Don’t Know	3680 (8.6)	1342 (7.1) *	2338 (9.8) *
	Refused	24 (0.1)	5 (0.0) *	19 (0.1) *

* Chi-square test: *p* < 0.001. HPV: human papillomavirus. Subsample A had adequate provider-reported vaccination records as well as the parent-reported vaccination information. Subsample B had only the parent-reported vaccinated information.

**Table 2:** The false-negative and false-positive cases of the parent-reported responses (Method 1) against the provider-reported vaccination records (Method 2) at each level of socioeconomic status in Subsample A (N = 18,788).

Socioeconomic Status Indicator	Level or Category	Frequency (%)		
		False Negative (A)	False-Positive (B)	A or B
Mother’s education	<12 years	277/1405 (19.7) **	88/373 (23.6) *	365/1778 (20.5) **
	12 years	280/1906 (14.7) **	151/774 (19.5) *	431/2680 (16.1) **
	>12 years, non-college graduate	365/3325 (11.0) **	281/1596 (17.6) *	646/4921 (13.1) **
	College graduate	566/6800 (8.3) **	542/2609 (20.8) *	1108/9409 (11.8) **
Annual family income	Above poverty level, >USD 75 K	589/7139 (8.3) **	557/2922 (19.1) *	1146/10,061 (11.4) **
	Above poverty level, ≤USD 75 K	427/3677 (11.6) **	312/1587 (19.7) *	739/5264 (14.0) **
	Below poverty level	405/2146 (18.9) **	161/657 (24.5) *	566/2803 (20.2) **
	Unknown	67/474 (14.1) **	32/186 (17.2) *	99/660 (15.0) **

Chi-square test: ** *p* < 0.001, * *p* < 0.05. Also see Figure 2.

**Table 3:** The proportion of HPV-unvaccinated teens with the parent-reported vaccination response (Method 1: “No” responses) in the whole sample (N = 42,668).

Socioeconomic Status Indicator	Level or Category	Question: Has Your Teen Ever Received Any Human Papillomavirus Shots?			
		Yes (59.2%)	No (32.1%)	Don’t Know (8.6%)	Refused (0.1%)
		n = 25,263	n = 13,701	n = 3680	n = 24
Mother’s education	<12 years	1949 (47.5%)	1273 * (31.0%)	884 (21.5%)	1 (0.0%)
	12 years	3587 (54.5%)	2244 * (34.1%)	750 (11.4%)	4 (0.1%)
	>12 years, non-college graduate	6484 (57.9%)	3972 * (35.5%)	736 (6.6%)	7 (0.1%)
	College graduate	13,243 (63.7%)	6212 * (29.9%)	1310 (6.3%)	12 (0.1%)
Annual family income	Above poverty level, >USD 75 K	13,691 (62.5%)	6849 * (31.3%)	1338 (6.1%)	13 (0.1%)
	Above poverty level, ≤USD 75 K	6854 (57.3%)	4049 * (33.9%)	1046 (8.8%)	5 (0.0%)
	Below poverty level	3178 (54.0%)	1848 * (31.4%)	858 (14.6%)	1 (0.0%)
	Unknown	1540 (52.4%)	955 * (32.5%)	438 (14.9%)	5 (0.2%)

Chi-square test for “No” responses vs. the other three responses (“Yes”, “Don’t Know”, or “Refused”) according to socioeconomic status level. * *p* < 0.001. HPV: human papillomavirus.

**Table 4:** The proportion of HPV-unvaccinated teens with the parent-reported vaccination response (Method 1: “No” responses) in Subsample A (N = 18,788).

Socioeconomic Status Indicator	Level or Category	Question: Has Your Teen Ever Received Any Human Papillomavirus Shots?			
		Yes (64.3%)	No (28.5%)	Don’t Know (7.1%)	Refused (0.0%)
		n = 12,086	n = 5355	n = 1342	n = 5
Mother’s education	<12 years	932 (52.4)	491 * (27.6)	354 (19.9)	1 (0.1)
	12 years	1600 (59.7)	812 * (30.3)	267 (10.0)	1 (0.0)
	>12 years, non-college graduate	3094 (62.9)	1581 * (32.1)	244 (5.0)	2 (0.0)
	College graduate	6460 (68.7)	2471 * (26.3)	477 (5.1)	1 (0.0)
Annual family income	Above poverty level, >USD 75 K	6805 (67.6)	2788 * (27.7)	464 (4.6)	4 (0.0)
	Above poverty level, ≤USD 75 K	3278 (62.3)	1572 * (29.9)	414 (7.9)	0 (0.0)
	Below poverty level	1627 (58.0)	809 * (28.9)	366 (13.1)	1 (0.0)
	Unknown	376 (57.0)	186 * (28.2)	98 (14.8)	0 (0.0)

Chi-square test for “No” responses vs. the other three responses (“Yes”, “Don’t Know”, or “Refused”) according to socioeconomic status level. * *p* < 0.001. Also see Figure 3. HPV: human papillomavirus.

**Table 5:** The proportion of HPV-unvaccinated teens with the provider-reported vaccination record (Method 2: “No” vaccination) in Subsample A (N = 18,788).

Socioeconomic Status Indicator	Level or Category	Provider-Reported Vaccination	
		Yes (71.5%)	No (28.5%)
		n = 13,436	n = 5352
Mother’s education	<12 years	1405 (79.0)	373 * (21.1)
	12 years	1906 (71.1)	774 * (28.9)
	> 12 years, non-college graduate	3325 (67.6)	1596 * (32.4)
	College graduate	6800 (72.3)	2609 * (27.7)
Annual family income	Above poverty level, >USD 75K	7139 (71.1)	2922 * (29.0)
	Above poverty level, ≤USD 75K	3677 (69.9)	1587 * (30.1)
	Below poverty level	2146 (76.6)	657 * (23.4)
	Unknown	474 (71.8)	186 * (28.2)

Chi-square test: * *p* < 0.001. Also see Figure 3. HPV: human papillomavirus.

**Table 6:** The multivariate odds ratios (95% confidence intervals) for HPV-unvaccinated teens (Method 1: parental reports; Method 2: provider records) according to socioeconomic status after controlling for age, sex, race/ethnicity, and region in the whole sample (N = 42,668) and Subsample A (N = 18,788).

		Odds Ratio (95% Confidence Interval) for HPV-Unvaccinated Teens		
Socioeconomic Status Indicator	Level or Category	In the Whole Sample (Method 1)	In Subsample A (Method 1)	In Subsample A (Method 2)
Mother’s education	<12 years	1.11 (1.03–1.20)	1.12 (0.99–1.27)	0.81 (0.71–0.92)
	12 years	1.24 (1.17–1.32)	1.25 (1.14–1.38)	1.13 (1.02–1.24)
	>12 years, non-college graduate	1.29 (1.22–1.35)	1.33 (1.23–1.44)	1.27 (1.18–1.37)
	College graduate	1.00	1.00	1.00
Annual family income	Above poverty level, >USD 75 K	1.00	1.00	1.00
	Above poverty level, ≤USD 75 K	1.14 (1.08–1.19)	1.12 (1.04–1.21)	1.10 (1.02–1.18)
	Below poverty level	1.05 (0.98–1.12)	1.10 (1.00–1.21)	0.84 (0.76–0.94)
	Unknown	1.10 (1.01–1.19)	1.07 (0.90–1.28)	1.06 (0.89–1.27)

HPV: human papillomavirus.

## Data Availability

The NIS-Teen public-use data file is located at http://www.cdc.gov/vaccines/imz-managers/nis/datasetsteen.html. (Accessed on 4 April 2021).
